# BMP signaling and its paradoxical effects in tumorigenesis and dissemination

**DOI:** 10.18632/oncotarget.12151

**Published:** 2016-09-20

**Authors:** Lijie Zhang, Yingnan Ye, Xinxin Long, Pei Xiao, Xiubao Ren, Jinpu Yu

**Affiliations:** ^1^ Department of Immunology, Tianjin Medical University Cancer Institute and Hospital, National Clinical Research Center of Caner, Key Laboratory of Cancer Prevention and Therapy, Clinical Research Center for Cancer, Key Laboratory of Cancer Immunology and Biotherapy, Tianjin, P. R. China; ^2^ Cancer Molecular Diagnostic Center, Tianjin Medical University Cancer Institute and Hospital, National Clinical Research Center of Caner, Key Laboratory of Cancer Prevention and Therapy, Clinical Research Center for Cancer, Tianjin, P. R. China

**Keywords:** BMP signaling pathway, tumorigenesis, epithelial–mesenchymal transition, cancer stem cell

## Abstract

Bone morphogenetic proteins (BMPs) play important roles in embryonic and postnatal development by regulating cell differentiation, proliferation, motility, and survival, thus maintaining homeostasis during organ and tissue development. BMPs can lead to tumorigenesis and regulate cancer progression in different stages. Therefore, we summarized studies on BMP expression, the clinical significance of BMP dysfunction in various cancer types, and the molecular regulation of various BMP-related signaling pathways. We emphasized on the paradoxical effects of BMPs on various aspects of carcinogenesis, including epithelial–mesenchymal transition (EMT), cancer stem cells (CSCs), and angiogenesis. We also reviewed the molecular mechanisms by which BMPs regulate tumor generation and progression as well as potential therapeutic targets against BMPs that might be valuable in preventing tumor growth and invasion.

## INTRODUCTION

BMPs are important cytokines belonging to the Transforming Growth Factor (TGF)-β superfamily, which also includes TGF-βs, activins, inhibins, nodal, and myostatin [1, 2]. They were first described by Marshall R. Urist in the 1960s, when he suggested the presence of osteoinductive molecules in demineralized bone matrix extracts, but further gene identification only occurred in the late 1980s [3]. Thus far, more than 20 kinds of BMP ligands have been identified in humans. Based on the disparity of their sequences and functions, these ligands have been divided into at least four subgroups: BMP-2/-4 group; BMP-5/-6/-7/-8 group (osteogenic protein-1 [OP-1] group); BMP-9/-10 group; and BMP-3, -13, -11, -12, -14, and -15 group [4]. Among these ligands, the first three groups were profoundly studied in tumors and have been reported to participate in tumorigenesis and dissemination.

Several reviews have illustrated the rough backbone of the BMP signaling pathways [1, 5]. Briefly, BMP ligands bind to two receptor types (type I and type II) to form a heterotetrameric complex, which then binds to and phosphorylate the receptor-activated (R)-SMADs. The activated R-SMADs bind to the common SMAD (Co-SMAD) to form a complex, which translocates to the nucleus along with a number of transcription factors to drive the target genes expression [6-8]. In addition to the canonical SMAD pathway, BMPs activate the non-canonical SMAD pathways, such as phosphatidylinositol 3-kinase (PI3K)/AKT, mitogen-activated protein kinase (MAPK), nuclear factor kappa B (NF-κB), and Janus kinase/signal transducer and activator of transcription (JAK/STAT) signaling pathways, which form a complex network of molecular signals regulating a multitude of processes throughout the body [5, 9, 10].

BMPs were originally reported to induce bone and cartilage formation, which exhibit a wide range of biological effects on various cell types. BMPs play important roles in embryonic and postnatal development by regulating cell differentiation, proliferation, motility, and survival, thus maintaining homeostasis in different organs and tissues [6, 11]. Recently, more evidence demonstrated that BMPs participate in cancer development and progression.

## PARADOXICAL EFFECTS OF BMP SIGNALING ON TUMORIGENESIS AND DISSEMINATION

Studies have shown that BMPs display significantly higher expression in tumors, which have been used as new biomarkers for the prognosis of cancer patients. In hepatocellular carcinoma (HCC), several BMPs (BMP-4, -6, -7, -8, -9, -10, -11, -13, and -15) revealed enhanced expression levels [12]. In advanced non-small cell lung cancer, serum increased the BMP-2 level, and the advanced clinical stages were significantly correlated with poor prognosis, which can be regarded as an independent negative predictor for the prognosis of patients [13]. BMP-4 upregulation is closely associated with shorter patients' overall and disease-free survival, which serves as a novel marker for predicting the recurrence and prognosis of HCC patients after surgery [14]. In addition, high BMP-7 expression could be a useful predictive marker of poor prognosis in patients with lung cancer [15, 16], esophageal squamous cell carcinoma [17], colorectal cancer [18], and clear cell renal carcinoma [19].

The aberrant expression of BMPs is correlated with the proliferation, differentiation, and apoptosis of cancer cells and thus may be regarded as an oncogene. BMP-9 promotes the cell proliferation of ovarian cancer [20]. In addition, BMP-9 triggered the phosphorylation of SMAD1, 5, and 8 and the overexpression of inhibitors of DNA binding 1 (Id1), thereby promoting a proliferative response and exerting a remarkable anti-apoptotic function in HCC cells [21]. BMP-9 also led to an observable alteration in cell cycle regulator expression, including cyclinD1 protein upregulation and the downregulation of CDK-interacting protein p27 expression. Both events are involved in the progression from the G0/G1 phases toward the S-phase of the cell cycle [22, 23]. The BMP-downstream signaling pathway, such as SMAD, has a role in tumor development and metastasis. Reportedly, SMAD1, 5, and 8 promote tumors. For instance, SMAD5 expression is inversely correlated with the prognosis of serious ovarian cancer patients, and BMP-2 stimulated cellular proliferation by inducing phosphorylated SMAD5 (pSMAD5) translocation into the nucleus in ovarian cancer cells [24].

Aside from these impacts on tumorigenesis, BMP signaling is involved in the invasion and migration processes, which are prerequisites to metastatic spread. BMPs significantly promoted tumor migration by affecting the extracellular matrix (ECM) environment, such as integrin and matrix metalloproteinases (MMPs), which is a crucial factor in tumor migration. BMP-7 upregulates integrin avb3 expression, thereby inducing the migration activity in human chondrosarcoma cells [25]. BMPs accelerate pancreatic cancer cell invasiveness, which involves MMP-2 upregulation [26]. Furthermore, BMP-2-induced phosphorylation of SMAD2/3 promotes epithelial-mesenchymal transition (EMT) and induced cell invasion and migration in breast and pancreatic cancer cells [27].

However, some data revealed an opposite role of BMP signaling in tumors. BMP-10 was downregulated in gastric cancer samples [28]. BMP-6 expression was also absent in breast cancer tissues and might suppress breast cancer metastasis [29]. The inhibitory role of BMPs in tumorigenesis and dissemination has been widely reported in previous studies. For instance, BMP-2 and BMP-7 function as potent tumor suppressors in gastric carcinoma, renal cell carcinoma, lung and colorectal cancer, and osteosarcoma, in which BMPs suppress tumor growth by reducing the gene expression of tumorigenic factors and inducing the differentiation of cancer stem cells (CSCs) [30-33]. In HCC, BMP-2 induces apoptosis and plays an inhibitory role by virtue of their ability to increase the expression of the pro-apoptotic proteins caspase-3 and cleaved caspase-3 [34]. Correspondingly, BMP-4 and BMP-9 were also found to be potential anticancer agents in breast cancer [35]. BMP-4 causes a decline in granulocyte colony-stimulating factor (G-CSF) secretion, thereby reducing the number and activities of myeloid-derived suppressor cells (MDSCs) [36]. BMP-9 inhibits the bone metastasis of breast cancer cells by downregulating connective tissue growth factor expression [37]. BMP-9 also prevents the growth of prostate cancer cells by inducing tumor apoptosis, which is related to the upregulation of prostate apoptosis response-4 [38]. In other solid tumors, BMP-4 paraclinically inhibits tumor angiogenesis via the induction of thrombospondin-1 (TSP-1) [39]. At times, SMADs in the BMP signaling pathway could prevent tumor progression. The knockout of SMAD1 and SMAD5 in somatic cells of male and female gonads promotes metastatic granulosa cell tumorigenesis in mice, which implicated SMAD1 and SMAD5 as critical tumor suppressors [40]. In HCC, the inhibition of the BMP-4/SMAD1 signaling has been reported to suppress tumor migration, invasion, and EMT [41].

In conclusion, BMPs are described as both stimulator and inhibitor in different cancers; thus, we cannot simply define BMPs as oncogenes or anti-oncogenes. Collectively, the aforementioned evidence indicated that the effects of BMP signaling on tumor progress depends on the cell types and the tumor microenvironment. Therefore, in the current study, we reviewed recent studies focusing on BMP bilateral effects in tumorigenesis and the underlying signaling pathways regulating the paradoxical dilemmas.

## BILATERAL ROLES OF BMP SIGNALING IN CELLULAR EVENTS OF CARCINOGENESIS

Considering that BMPs simultaneously displayed both tumor-promoting and tumor-inhibiting effects, we must emphasize on the disparity of biological behavior and molecular events along the BMP signaling to disclose the underlying mechanisms involved in such paradoxical biological behaviors. Therefore, we focus on the different aspects during tumorigenesis and metastasis, including EMT, CSCs, and angiogenesis.

### BMP signaling and EMT

EMT is primarily defined as a phenotypic conversion that facilitates embryonic development and wound healing in physiological processes. Moreover, the acquisition of the EMT phenotype is related to fibrosis and tumor progression in certain pathological processes [42, 43]. Undergoing EMT, the epithelial cancer cells go through multiple changes, which mainly include the suppression of epithelial characteristics and the acquisition of a mesenchymal phenotype at the invasive front [44-46]. These hallmarks of EMT in cancers include the loss of E-cadherin expression, reduction of tight junction proteins [such as zona occludens-1 (ZO-1)] and cytokeratin, and increase of mesenchymal markers, such as vimentin, fibronectin, and N-cadherin [47, 48].

Multiple transcriptional factors and molecules take part in the tumor EMT procedure. Among them, the Snail superfamily (particularly Snail 1 and Snail 2), the basic helix-loop-helix family (such as Twist), and two Zeb factors (Zeb1 and Zeb2) regulate the expression of various epithelial and mesenchymal genes and thus affect the biological processes of cytoskeletal reorganization, extracellular matrix remodeling, and cell movements during EMT [49, 50]. For instance, Zeb1 and Zeb2 lead to E-cadherin repression, cause dramatic morphological transition of cells, and enhance migration and invasion during cancer progression [51]. Overexpression of Twist causes E-cadherin downregulation and vimentin upregulation to induce cellular morphological changes, expands the stem cell population, and promotes cell migration and invasion [52]. β-catenin and ZO-1 induced cytoplasmic/nuclear relocalization is a common process for EMT associated with tumor invasion [53].

EMT is associated with cancer invasion and metastasis in various tumor types, and the acquisition of mesenchymal features is related to the enhancement of tumor invasive capacity during cancer progression [54-56]. In breast cancer, the downregulation of the epithelial marker E-cadherin and upregulation of the mesenchymal markers, N-cadherin and vimentin, were positively correlated with the high aggressiveness and rapid spread of cancer [57]. Consistently, EMT plays a crucial role in the early steps of metastasis in HCC where the low E-cadherin expression and high vimentin expression were closely associated with high-grade tumor vascular invasion [58, 59]. The mesenchymal to epithelial transition (MET) is the reverse procedure of EMT, during which cell motility dramatically decreases [46]. MET attenuates the malignancy of cancer cells in squamous cell carcinoma [60]. Similarly, the proliferation, migration, and invasion of gastric carcinoma cells are suppressed during MET [61]. Therefore, MET plays an inhibitory role in tumor metastasis.

Observable EMT features of cancer cells could be induced by BMPs via SMAD and non-SMAD signaling pathways, which promote tumor invasion and metastasis in vitro and in vivo [62-64]. For instance, in breast epithelial and ovarian cancer cells, BMP-4 could induce EMT by decreasing E-cadherin, increasing N-cadherin, disrupting the polarity of ZO-1, and inducing transcription factors, Slug and Snail, to facilitate tumor progression [65, 66]. The reduction of E-cadherin and ZO-1 and an induction of vimentin and Snail expression have also been observed after the BMP-9 treatment in HCC [67]. Similarly, BMP-2 and BMP-7 were reported to promote the characteristic morphologic conversion of EMT in gastric and prostate cancer cells [62, 63].

Aside from the positive effects on EMT, literature has shown the opposite roles of the BMP signaling in inhibiting EMT-related metastasis of cancer. In melanoma cells, BMP-7 induced MET, a process opposite to EMT at the primary tumor site, thereby leading to metastasis inhibition [68]. BMP-7 also inhibited cholangiocarcinoma cell migration by suppressing TGF-β-mediated Twist expression, which is an important EMT transcription factor [69]. Accordingly, the effects of BMPs reversing EMT have been determined in breast cancer cells where BMP signaling induced E-cadherin expression and limited cancer cell metastatic potential by repressing EMT-activator Zeb1genes [70].

### BMP signaling and CSCs

CSCs were described as immortal, possessing self-renewal capacity [71, 72], highly tumorigenic [73], and resistant to conventional chemotherapies [74]. They were initially reported in acute myeloid leukemia [75]. Recent reports verified the CSCs' presence in breast [76], colon [77], prostate [78], skin [79], liver [80], stomach [81], lung [82], and brain cancers [83]. BMP signaling participated in CSC-related tumor maintenance and progression by influencing the CSCs' functional properties, such as self-renewal, chemo-resistance, and tumor-initiating capacities [84]. For instance, BMP signaling hyper-activated and accelerated the amplification of tumor stem cell populations during the initiation and progression of breast cancer and oral squamous cell carcinoma [85, 86]. BMP-2 enhanced the motility and invasiveness of colon cancer cells by inducing CSC proliferation [87]. In ovarian cancer, human carcinoma-associated mesenchymal stem cells could increase the number of CSCs and promote the chemotherapy resistance of ovarian cancer by activating the BMP-4/Hedgehog signaling pathway [88]. BMP signaling could crosstalk with several known stem cell pathways in cancer, such as the Notch pathway and the Wnt pathway. In colorectal cancer, Notch signaling interacts with the BMP signaling in CSC regulation, in which Notch-1 expression increased along with the upregulation of multiple EMT/stemness-associated molecules CD44, Slug, and SMAD3 that led to a more aggressive phenotype [89].

By contrast, other reports demonstrate that BMP signaling diminishes the CSC pool. In colorectal cancer and nervous system tumors, BMP-4 promotes the differentiation, apoptosis, and chemo-sensitization of CSCs and restricts the self-renewal capacity of CSCs, which plays an inhibitory role on tumor progression [90-92]. Furthermore, other known stem cell pathways, such as the Wnt/β-catenin signaling, have been found to crosstalk with the BMP signaling in CSC regulation. For example, intestinal adenoma cells produce BMP-4 to counteract the Wnt/β-catenin signaling-related CSC-like traits, such as losing self-renewal capacity and initiating irreversible cellular differentiation [93]. In human renal cancer, BMP-2 suppresses the growth of aldehyde dehydrogenase (ALDH)^+^ cells, downregulates the expression of embryonic stem cell markers, and inhibits renal CSC migration [94]. BMP-2/-7 heterodimer, the most efficient stimulator of BMP signaling, diminishes the ALDH^hi^/CD44^hi^/CD24^low^ CSC pool and effectively reduces the activation of TGF-β-driven SMAD signaling pathway, thereby inhibiting tumor invasion in breast cancer [95]. In prostate cancer, BMP-7 increases the expression of cell cycle inhibitor p21 and metastasis suppressor gene NDRG1 (N-myc downstream-regulated gene1) to induce CSC senescence [96]. In glioblastoma, BMP-7 reduces cell growth, inhibits sphere formation, and decreases self-renewal capacity via canonical SMAD1, 5, and 8 signaling [97]. Similarly, a BMP-7 variant (BMP-7v) represses the proliferation of stem-like cells and the expression of stem cell markers and enhances the expression of differentiation marker in glioblastoma [98]. The BMP-mediated repression on CSCs was also found in head and neck squamous cell carcinoma (HNSCC), in which inhibiting BMP signaling potentiated the long-term survival of HNSCC CSCs [99].

### BMP signaling and angiogenesis

Tumor lymphatic and vascular angiogenesis has a key role in cancer development and progression by providing a faster and easier route for cancer cells to spread to other body parts. The tumor-associated angiogenesis is affected by multiple factors, among which BMPs are considered as important modulators. BMPs participate in angiogenesis not only by directly regulating the functions of vascular endothelial cells but also by indirectly influencing the expression of multiple angiogenic factors [100, 101]. For instance, BMPs upregulate the expression of vascular endothelial growth factor (VEGF) in both prostate cancer cells and osteoblasts, thereby induce brain metastases [102]. BMP-9 and BMP-10 increase gene expression along the Notch signaling pathway in vascular endothelial cells, thereby coordinating postnatal vascular remodeling [103].

Recently, increasing evidence indicated that BMP signaling promotes tumor angiogenesis. BMP co-receptor repulsive guidance molecule b (RGMb) was upregulated in vascular endothelial cells after hepatocyte growth factor (HGF) stimulation, which was combined with BMP-7 to induce angiogenesis in breast and prostate cancers [104]. In human dermal microvascular endothelial cells, BMP-2 induces Id1 expression and cooperates with VEGF signaling to promote angiogenesis in murine breast cancer xenograft models [105]. Another case for the pro-angiogenic role of the BMP signaling during cancer development came from colorectal cancer studies in which miR-885-3p inhibited the growth of HT-29 colon cancer cell xenografts by disrupting angiogenesis via targeting BMPRIA and blocking the activation of the BMP/SMAD/Id1 signaling [106].

However, most data implied the paradoxical role of BMP signaling in tumor angiogenesis. For example, BMP-4 was downregulated in high endothelial venules of lymph nodes draining metastatic tumors [107]. In multiple myeloma, BMP-6 induces cell apoptosis, inhibits angiogenesis, and causes growth suppression [108]. BMP-9 inhibits basic fibroblast growth factor (bFGF)-induced proliferation and migration of bovine aortic endothelial cells and represses VEGF-stimulated angiogenesis in glioblastoma [109]. BMP-9/ALK1-induced Crossveinless 2 and matrix Gla protein inhibits angiogenesis by limiting proliferation, tube formation, and expression of VEGF of endothelial cells [110]. In addition, BMP-9 suppresses lymphatic vessel formation and restrains sprouting angiogenesis and blood circulation [111, 112].

## MOLECULAR PATHWAYS REGULATED BY THE BMP SIGNALING PATHWAY

As previously described, BMP signaling plays a paradoxical role in cancer by affecting various features of carcinogenesis, which include EMT, CSCs, and angiogenesis. Increasing evidence has focused on multiple molecular events regulating BMP-induced biological processes in cancer and demonstrated that BMP signaling influenced the tumorigenesis and dissemination by modulating either the canonical SMAD or the non-canonical SMAD signaling pathways [113].

In skull-based chordomas, the BMP-4/SMAD signaling pathway upregulation was reported to be the dominant molecular mechanism of chordoma pathogenesis, which indicated poor clinical outcome [114]. Growth and Differentiation Factor (GDF)-9, a member of the BMP ligand family, promotes the adhesive and motile capacity of cancer cells by upregulating focal adhesion-associated proteins FAK and paxillin via the SMAD-dependent pathway, which implied pro-tumorigenic effects of the canonical BMP/SMAD signaling pathway in prostate cancer [115].

In addition to the canonical SMAD signaling pathways, the non-canonical SMAD signaling pathways participate in BMP-related cancer development and progression. BMPs activate the PI3K/AKT signaling pathways in gastric cancer, chondrosarcoma, and pancreatic cancer [116-118]. The PI3K/AKT pathway, a major cascade-promoting cell migration and invasion, could be activated by BMP-2 in gastric and pancreatic cancer cells, which can dramatically enhance the phosphorylation level of AKT protein. The blockage of the PI3K/AKT pathway using specific inhibitor LY294002 significantly reverts EMT and inhibits BMP-2-induced motility and invasiveness [62]. In addition, MAPK/ERK signaling pathway is another important regulator in cell migration and invasion, which was promoted by BMPs in which the inhibition of the RAF/MEK/ERK cascaded along the MAPK pathway reduced BMP7-induced motility and migration that subsequently led to cell apoptosis in prostate cancer cells [63]. Furthermore, BMP-2 enhances the phosphorylation of IκBα and the nuclear translocation of NF-κB in gastric and prostate cancer cells [119, 120]. Similarly, exogenous BMP-7 activates the c-Src/PI3K/AKT/IKK/NF-κB signaling pathway, thus resulting in the trans-activation of avβ3 integrin expression, which promotes tumor progression in human chondrosarcoma cells [25]. BMP-2 induces EMT and promotes colon cancer cell migration and invasion by increasing STAT3-mediated tumor stemness [87].

However, some studies pointed out that BMPs could exert an antitumor effect by blocking non-canonical signaling pathways in certain cancers. BMP-9 prevented the proliferation of HER2-positive breast cancer cells by inactivating ERK1/2 protein and repressing the PI3K/AKT signaling pathway [35]. In gastric cancer, PI3K/AKT pathway inhibition was involved in the tumor-suppressor effects of BMP-9 [121]. Similar to BMP-9, BMP-2 inhibits the growth and migration of HCC cells by attenuating the PI3K/AKT signaling pathway [34]. In addition, BMP-2 causes cell cycle arrest at the G1 phase and induces the apoptosis of myeloma cells by STAT3 inactivation [122]. Additionally, BMP-4 reduces the secretion of G-CSF and decreases the number and activities of MDSCs by counteracting NF-κB activity in tumor cells [36].

Collectively, BMPs play bidirectional and paradoxical effects on cancer development and invasion both at the molecular and cellular levels, in which the dysregulation of both the canonical and non-canonical SMAD pathways, including PI3K/AKT, MAPK/ERK, NF-κB, and STAT3 pathways, produces absolutely opposite influences on cell proliferation, apoptosis, migration, and invasion by affecting tumor EMT, generation and amplification of CSCs, and angiogenesis development.

## THERAPEUTIC APPROACHES AGAINST BMP SIGNALING IN CANCER

Considering the extensive involvement of BMP signaling in carcinogenesis and dissemination, target therapy against BMPs and their receptors is a promising approach for cancer treatment. However, given that BMPs produce paradoxical effects in different types of cancer, the personalized treatment against BMPs should be discussed considering the characteristics of cancer cells, the disparity of components in tumor microenvironment, and the interaction among different signal pathways in each study model to achieve the best therapeutic efficiency.

### Therapy against BMPs in cancer

BMPs are the hot topic of target therapy where some recombinant human BMPs, such as BMP-2 and BMP-7, have been used in orthopaedic and dental surgery [123-127]. However, clinical applications of BMPs in cancers are fewer because of their paradoxical effects on carcinogenesis and dissemination. Some studies have proposed potential applications for BMPs in cancer therapy.

In certain cancer types, BMPs that play pro-tumorigenic effects have been identified as novel prognostic biomarkers and potential therapy targets for cancer diagnosis and treatment. BMP-4, BMP-6, BMP-7, and BMP-9 are being proposed as biomarkers for HCC recurrence prediction and prognosis [14, 21, 128-131]. Some inhibitors that target BMPs have been proposed to be used in these cancers. Berberine, a natural alkaloid with important antitumor activities, has exerted inhibitory effects on the migratory and invasive abilities of highly metastatic prostate cancer cells by downregulating BMP-7 [132]. The active compounds tetramethylpyrazine, which was extracted from a Chinese medicinal plant, and heparan sulfate mimetic WSS25 respectively inhibited angiogenesis and tumor growth of lung and hepatocellular cancer by blocking BMP/SMAD/Id-1 signaling [133, 134]. Coleusin factor, an inhibitor targeting BMP-2, exerted its anticancer effects on osteosarcoma by inducing osteoblast differentiation [135]. Moreover, phosphoprotein Spp24 secreted by BMP binding protein diminished BMP-2-initated tumor growth and thus resulted in significant apoptosis of cancer cells, which would be developed into a new therapeutic agent for clinical applications [136].

By contrast, BMPs function as tumor suppressors in some cancer types. BMP-4, BMP-6, and BMP-9 have been reported to inhibit metastasis in breast cancer. BMP-2 plays a key inhibitory role in governing the proliferation and aggressive features of human cancer cells in HCC and colorectal carcinoma. Therefore, therapies based on BMP signaling activation may offer a novel treatment strategy for these cancer types [29, 33, 37]. Recombinant BMP ligand domains, which are being used as efficient agents for the repair of bony defects in preclinical and clinical studies of orthopaedic and maxillofacial surgery, are currently being tested for their therapeutic feasibilities in cancer. For instance, recombinant human (rh) BMP-7 exerted antineoplastic effects in HNSCC and rhBMP-2 was applied to treat osteosarcoma by increasing caspase-3 and Bax-mediated cell apoptosis in cancer [137, 138].

**Figure 1:**
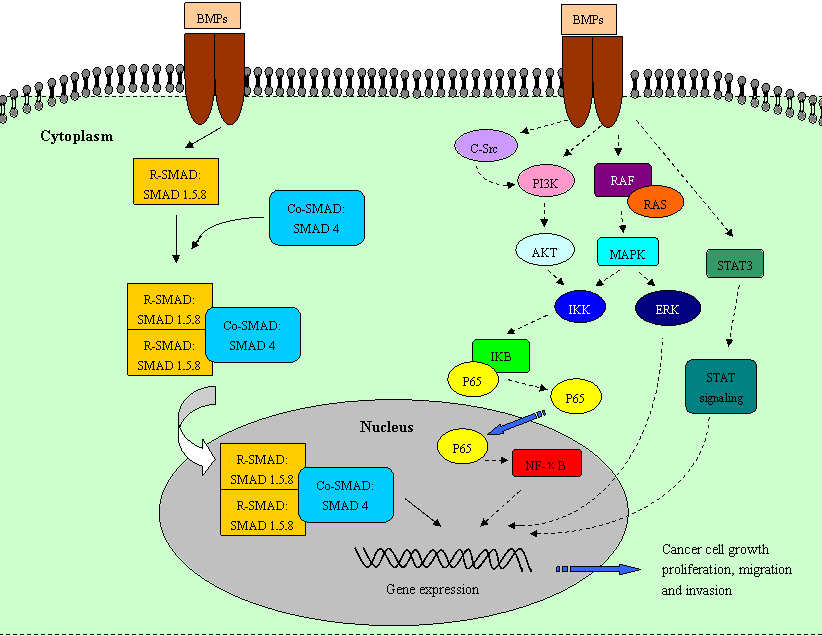
Relevant pathways regulate the paradoxical effects of BMP signaling in tumors BMP signaling influences cancer cell progression through canonical SMAD and non-SMAD signaling pathways. The canonical SMAD-signaling pathway functions as a stimulator for cancer progression. However, non-SMAD signaling pathways, including PI3K/AKT, MAPK, NF-κB, and JAK/STAT signaling pathways, play bidirectional roles in various cancers. BMP signaling can cause the activation and inactivation of these non-SMAD signaling pathways to promote or suppress tumor progression.

### Therapy against BMP receptors in cancer

BMP receptors have also been studied as target candidates for cancer therapy. Activin receptor-like kinases 1 (ALK1), a BMP receptor type I, has been confirmed as a target for anti-angiogenesis in cancer. Both ALK1 neutralizing antibodies and soluble ALK1 extracellular domain/Fc fusion protein (ALK1-Fc) attenuate the BMP signaling activity [139]. For example, Dalantercept, a fusion product composed of extracellular domain of ActRIIA and IgG-Fc fragment, was proposed as a novel anti-angiogenic therapy for treating a variety of cancers both in preclinical and clinical studies [140, 141].

Furthermore, the therapeutic effects of specific inhibitors against BMP receptor kinases have been investigated in various cancer types. Dorsomorphin and its analogue LDN-193189, which are the original inhibitors of ALK1, produce a block in cell migration and increase survival in human epithelial ovarian cancer [142]. Another small molecule inhibitor K02288, a 2-aminopyridine compound targeting ALK1, could inhibit BMP-9 signal transduction and thus repress tumor angiogenesis in diffused intrinsic pontine glioma and other tumors [143]. EW-7197, a novel ALK5 kinase inhibitor, represses the activation of the SMAD/TGF-β signaling pathway, thereby preventing lung metastasis in mouse 4T1 mammary cancer models and prolonging the survival of 4T1-bearing mice [144]. Recently, a selectively small molecule inhibitor DMH1, which specifically antagonizes the intracellular kinase domain of ALK2, significantly reduces cell proliferation, promotes cell death, and decreases cell invasion in NSCLC, thereby providing a promising development of target therapeutic strategy for clinical applications [145].

**Table 1:** The list for BMP family members

		Known receptors		
Ligand	Gene locus	Type I receptors	Type II receptors	Functions
BMP-1	8p21.3			Extracellular matrix maintenance, chondrogenesis
BMP-2	20p12	ALK-2, ALK-3, ALK-6	BMPR-II, ActR-IIA, ActR-IIB	Osteoblast differentiation, bone and cartilage formation. Aretinoid mediator. Involved indorsoventral patterning, craniofacial and heart development
BMP-3	14p21.21	ALK-4	ActR-IIA, ActR-IIB	Bone formation
BMP-4	14q22-q23	ALK-2, ALK-3, ALK-5, ALK-6	BMPR-II, ActR-IIA	Fracture repair, Formation of teeth, limbs, lung, eye, and bone fromMesoderm, Dorsoventral patterning and craniofacial development
BMP-5	6p12.1	ALK-3	BMPR-II, ActR-IIA, ActR-IIB	Chondrogenesis
BMP-6	6p24-p23	ALK-2, ALK-3, ALK-6	BMPR-II, ActR-IIA, ActR-IIB	Involved in joint integrity, osteogenesis and chondrogenesis
BMP-7	20q13	ALK-2, ALK-3, ALK-6	BMPR-II, AMHR-II	Osteoblast differentiation, renal development/repair, eye and craniofacial development
BMP-8a	1p34.3	ALK-2, ALK-3, ALK-4, ALK-7	BMPR-II, AMHR-II	Osteogenesis, chondrogenesis and craniofacial development
BMP-8b	1p35-p32	ALK-3, ALK-6	BMPR-II, ActR-IIA, ActR-IIB	Osteogenesis, chondrogenesis and craniofacial development
BMP-9	10q11.22	ALK-1, ALK-2	BMPR-II, ActR-IIA, ActR-IIB	Chondrogenesis, nervous system, hepatogenesis and hepatic reticuloendothelial system development
BMP-10	2p13.3	ALK-1, ALK-3, ALK-6	ActR-IIA, ActR-IIB	Trabeculation of embryonic heart
BMP-11	12q13.2	ALK-3, ALK-4, ALK-5, ALK-7	BMPR-II, ActR-IIA, ActR-IIB	Mesodermal patterning and nervous system development
BMP-12	2p24.1	ALK-3, ALK-6	BMPR-II, ActR-IIA	Joint morphogenesis, Facilitates growth of ligament and tendon
BMP-13	8q22.1	ALK-3, ALK-6	BMPR-II, ActR-IIA, ActR-IIB	Joint morphogenesis, Facilitates growth of ligament and tendon
BMP-14	20q11.2	ALK-3, ALK-6	BMPR-II, ActR-IIA	Chondrogenesis, limb development, fracture healing and facilitates growth of tendon
BMP-15	Xp11.2	ALK-6		Oocyte and follicular development

This table shows the members of BMP family, gene locus, relative receptors and functions (if known).

## CONCLUSIONS AND PERSPECTIVES

In this review, we summarized studies regarding the paradoxical roles of BMP signaling in tumor generation and progression. The effects of BMP signaling on cancer are closely related to the pathological type, the tumor origin, the activation status of downstream signaling pathways, and the various factors in tumor microenvironment. BMP signaling plays a paradoxical effects on cancer development and progression by serving as either tumor promoters or tumor suppressors, which dramatically affects tumor EMT, stemness, and angiogenesis. Targeting BMPs and BMPRs were successful in preventing tumor growth and invasion in some preclinical and clinical studies, which implies a promising future on BMPs target therapy in cancer treatment.
